# Prophylactic cranial irradiation for extensive stage small cell lung cancer: a meta-analysis of randomized controlled trials

**DOI:** 10.3389/fonc.2023.1086290

**Published:** 2023-05-17

**Authors:** Ziyi Wang, Liang Chen, Lu Sun, Feng Cai, Qiwei Yang, Xiaohai Hu, Qiang Fu, Weiyang Chen, Peiwei Li, Wenya Li

**Affiliations:** ^1^ Department of Thoracic Surgery, The First Hospital of China Medical University, Shenyang, Liaoning, China; ^2^ Department of Radiation Oncology, The First Hospital of China Medical University, Shenyang, Liaoning, China

**Keywords:** PCI for extensive stage SCLC prophylactic cranial irradiation, small cell lung cancer, overall survival, meta-analysis, brain metastases, survival rates

## Abstract

**Background:**

Previous studies have demonstrated that prophylactic cranial irradiation (PCI) could reduce the risk of brain metastases and prolong the overall survival (OS) of patients with small cell lung cancer (SCLC). However, it remains controversial whether the efficacy and safety of PCI would be subjected to the different characteristics of patients with extensive stage of SCLC. This meta-analysis aims to evaluate the efficacy and safety of PCI in patients with extensive stage SCLC.

**Methods:**

PubMed, Embase, and the Cochrane Library were searched for relevant studies from inception to May, 2021. Hazard ratios (HRs) were used to measure the OS and progression-free survival (PFS), and relative risks (RRs) were employed to calculate the incidence of brain metastases, survival rate, and adverse events. Summary results were pooled using random-effect models.

**Results:**

There were 1215 articles identified, and 15 trials were included, with a total of 1,623 participants. Patients who received PCI did not result in significantly improved OS [HR=0.87, 95%CI (0.70, 1.08) *p*=0.417] and PFS [HR=0.81, 95%CI (0.69, 0.95) *p*=0.001], compared with those who did not receive PCI, while patients who received PCI had a significantly decreased incidence of brain metastases [RR=0.57, 95%CI (0.45, 0.74), *p*<0.001]. PCI group showed no improvements in 2-year (RR=1.03, *p*=0.154), 3-year (RR=0.97, *p*=0.072), 4-year (RR=0.71, *p*=0.101) and 5-year survival rates (RR=0.32, *p*=0.307), compared with non-PCI group, whereas the overall RR indicated that PCI was associated with a higher 1-year survival rate [RR=1.46, 95%CI (1.08, 1.97), *p*=0.013]. In addition, PCI treatment was shown to be associated with increased incidence of adverse events, including fatigue, dermatitis, anorexia, nausea, vomiting, malaise, and cognitive impairment.

**Conclusion:**

This meta-analysis suggests that PCI can reduce the incidence of brain metastases in extensive stage SCLC. Although PCI has no significant effect on the OS, it improves 1-year survival in patients with extensive stage SCLC. However, PCI does not significantly affect 2,3,4,5-year survival and may result in a significantly increased risk of adverse events.

## Introduction

Lung cancer is the leading cause of cancer-related mortality worldwide, with approximately 1.38 million deaths reported annually (1). Small cell lung cancer (SCLC) accounts for nearly 14% of primary lung cancer, and over 70% of SCLC patients are diagnosed at extensive-stage (2, 3). Extensive stage SCLC (ES-SCLC) is characterized by a higher risk of metastasis, more rapid doubling time, and earlier dissemination (4). Four to six courses of cisplatin-containing chemotherapy are the primary regimen widely used for patients with extensive stage SCLC, with considerable clinical response of 60 to 70%, while the median survival of patients with extensive stage SCLC remains poor at 9 months (5–7). Further, brain metastases could compromise the quality of life and survival time in patients with extensive stage SCLC in that many of the patients died of intra-cerebral progression (8). MRI is one of the best diagnostic methods for early brain metastases.

Prophylactic cranial irradiation (PCI) is widely applied for the prevention of early cancer dissemination to the uninvolved brain, since not all systemic chemotherapeutic drugs can cross the blood-brain barrier (BBB). Existing BBB-crossing systemic therapies that are available is not first-line drugs or only applicable for tumors with specific mutations (9). Several studies have already demonstrated that PCI was associated with a lower risk of brain metastases and longer survival time in patients with limited stage SCLC (6, 10–14). Patients with extensive stage SCLC have a shorter median survival time and a higher risk of brain metastases after chemotherapy than those with limited stage SCLC. Therefore, PCI should be recommended for these patients to reduce the incidence of brain metastases. However, the data on the efficacy and safety of PCI for patients with extensive stage SCLC remains limited and inconclusive. In this study, we reviewed the available randomized controlled trials (RCTs) to evaluate the efficacy and safety of PCI in patients with extensive stage SCLC, and we further explored its effects in the treatment for patients with specific characteristics.

## Methods

### Search strategy

This systematic review and meta-analysis was conducted and reported according to *the Preferred Reporting Items for Systematic Reviews and Meta-Analysis* (PRISMA) statement (15). PubMed, EMBASE, and the Cochrane Library databases were searched, from inception to May, 2021 for RCTs that conducted to evaluate the efficacy and safety of PCI for the treatment of extensive stage SCLC, without no language restrictions. Search items mainly included “small cell lung cancer” and “prophylactic cranial irradiation”. The reference lists of included studies were manually searched for potential eligible studies. Literature search was processed independently by 2 review authors. Any disagreement was settled by the primary author.

### Inclusion criteria

Studies meeting the following criteria were included:

(1) Types of study: RCT-design;
(2) Types of intervention: comparison between PCI and non-PCI;
(3) Types of participants: patients with extensive stage SCLC (If both limited and extensive stage patients were included in trials, only the data of the latter were extracted separately);
(4) Types of outcome: overall survival (OS), progression free survival (PFS), incidence of brain metastases, and survival rate at different follow-up periods.

Non-randomized studies and studies that only involved patients with limited stage SCLC were excluded.

### Quality assessment and data extraction

Quality assessment and data extraction were conducted by two review authors independently, and any disagreement was resolved by group discussion until a consensus was reached. The revised JADAD scale was applied to assess the quality of included studies, which is a comprehensive assessment tool and has been partially validated for evaluating the quality of RCTs in meta-analyses (16). The JADAD scale is based on 5 items: randomization (0-2), concealment of the treatment allocation (0-1), blinding (0-2), completeness of follow-up (0-1), and use of intention-to-treat analysis (0-1). Each item could be scored for 0 to 2, with a total score of 7. Study with a score of 4 or more would be regarded as high quality. Information was verified and adjudicated independently by a third author according to the original studies.

Data were extracted using a pre-designed form, containing name of the first author, publication date, country, sample size, characteristics of participants (mean age and gender distribution), follow-up duration, total dose/No. of fractions of PCI, OS, PFS, incidence of brain metastases, 1-, 2-, 3-, 4-, and 5-year survival rates, and any possible adverse events (all grades).

### Statistical analysis

Hazard ratio (HR) was applied as pooled statistic for survival data including OS and PFS, with the 95% confidence intervals (CI) provided. Relative risk (RR) and the 95%CI were used for dichotomous data, including incidence of brain metastases, 1-, 2-, 3-, 4-, and 5-year survival rates, and any possible adverse events. Random-effect model were applied to pool the effects (17, 18). Heterogeneity test was performed using Cochrane’s Q test and *I^2^* test. A *p* value less than 0.10 indicated statistical significance (19, 20). Sensitivity analysis was performed by removing each study one by one from the meta-analysis to assess its influence (21). Subgroup analysis was conducted for OS, PFS, incidence of brain metastases, 1-, 2-, 3-, 4-, and 5-year survival rates based on publication date (before vs after 2000), regions (Eastern vs Western), sample size (≥ 100 vs <100), mean age (≥ 60.0 vs <60.0), the proportion of males in the study (≥ 70.0% vs <70.0%), and quality of the studies (4-7 vs 1-4). The efficacy and safety of PCI according to the aforementioned factors were correlated with background treatment strategies, ethnicity, statistical power, patient characteristics, and strength of evidence. The *p* values between the subgroups were calculated using the Chi-square test and meta-regression (22). Publication bias was evaluated using the Egger`s test (23) and Begg`s test (24). All reported *p* values were two-sided and the values less than 0.05 were considered significant for all included trials. Statistical analyses were performed using STATA software (version 10.0 StataCorp, Texas, USA).

## Results

### Study selection and characteristics of included studies

**Figure 1:**
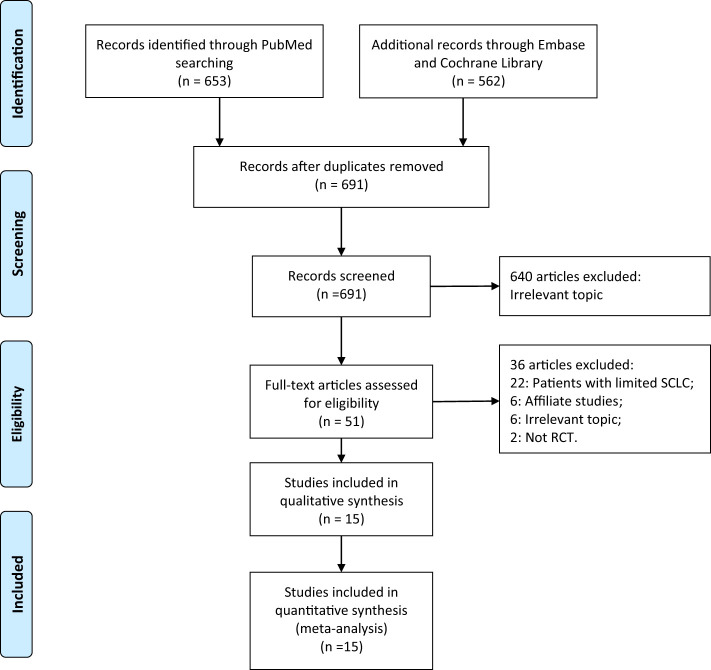
PRISMA flow diagram of study selection.

There were 1,215 articles identified, and 1,164 were removed after duplicate-checking and titles- and abstracts-reading. Full-texts of the remaining 51 articles were retrieved and read. We further excluded 22 studies that enrolled patients with limited stage SCLC, 6 studies reporting on the same populations, 2 non-RCT studies, and 6 studies focusing on other topics. After the detailed evaluation, 15 RCTs involving 1623 extensive stage SCLC patients were included (8, 9, 25–37). A manual search of the reference lists of these studies did not yield any additional eligible studies. The selection process of this study was presented in a PRISMA flowchart (**Figure 1**), and the baseline characteristics of included studies were shown in **Table 1**. Quality assessment was performed using the JADAD scale, with 1 study scoring for 5 (25), 8 studies scoring for 4 (8, 26, 27, 29, 33–36), and the remaining 6 for 3 (9, 28, 30–32, 37).

**Table 1:** Baseline characteristics of studies included in the systematic review and meta-analysis.

Study	Publication year	Country	Sample size	Mean age (years)	Percentage male (%)	Follow-up (months)	Total dose/No. of fraction	JADAD score
Slotman (8)	2007	UK	286	62.5	62.6	12.0	20-30 Gy/5-12	4
Takahashi (25)	2017	Japan	224	69.0	86.2	11.3	25 Gy/10	5
Ready (26)	2015	USA	85	60.0	44.7	12.0	25 Gy/10	4
Schild (9)	2012	USA	318	62.0	58.1	72.0	25-30 Gy/10-15	3
Shaw (28)	1994	USA	165	NA	NA	48.0	30-38 Gy/10-18	3
Laplanche (29)	1998	France	33	57.5	90.5	60.0	24-30 Gy/8-10	4
Gregor (30)	1997	UK	3	60.0	63.0	18.0	24-36 Gy/12-18	3
Arriagada (31)	1995	France	56	56.5	87.1	48.0	39 Gy/22	3
Ohonoshi (32)	1993	Japan	16	64.0	73.9	102.0	40 Gy/20	3
Aroney (33)	1983	Australia	10	62.0	NA	222.0	30 Gy/10	4
Danish/NCI (34)	1991	USA	12	59.0	NA	105.6	24 Gy/8	4
Wagner (35)	1996	USA	7	NA	NA	46.8	24 Gy/8	4
Belderbos (36)	2020	Netherlands	168	64	49	24.8	25 Gy/10	4
Rule (27)	2015	USA	155	72.8	59.4	60.0	30Gy/15	4
Salama (37)	2016	USA	85	60	44.7	24	25Gy/4-6	3

NA, Not applicable.

### OS and PFS

**Figure 2:**
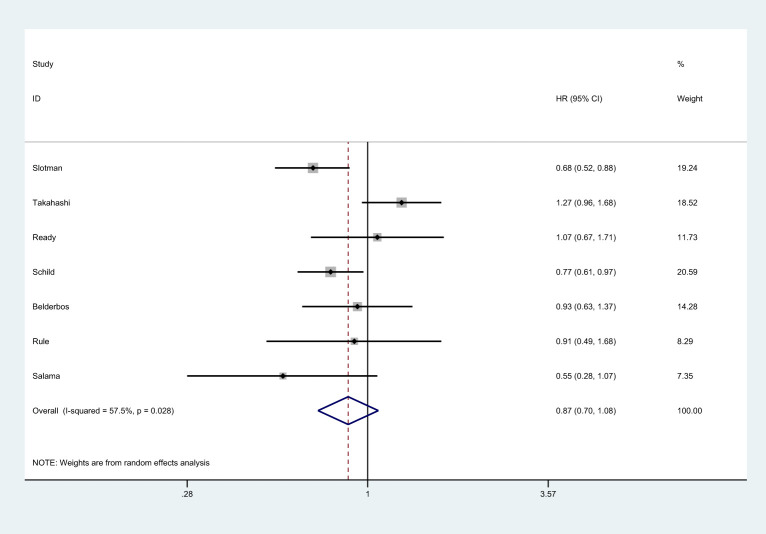
Effect of PCI on OS in patients with extensive SCLC.

There were 7 studies that reported OS (8, 25–27, 36, 37). There was no significantly statistical difference in the improvement of OS between PCI group and non-PCI group [HR=0.87, 95%CI (0.70, 1.08), *p*=0.417] (**Figure 2**). There was significant heterogeneity among the studies (I^2 ^= 57.5%; *p*=0.028). Sensitivity analysis showed that heterogeneity may come from Takahashi et al. (25) (**Supplemental 1**). Subgroup analysis indicated that PCI significantly improved OS in trials conducted in Western countries, studies with the proportion of males over 70.0%, and studies with low JADAD scores (**Supplemental 2**).

**Figure 3:**
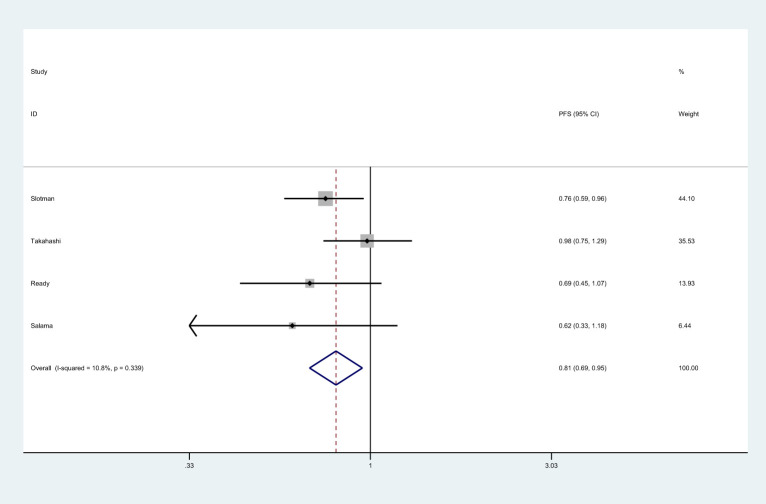
Effect of PCI on PFS in patients with extensive SCLC.

There were 4 studies that reported PFS (8, 25, 26, 37). Meta-analysis showed that patients in PCI group had longer PFS than those in non-PCI group [HR=0.81, 95%CI (0.69, 0.95), *p*=0.001] (**Figure 3**). No significant heterogeneity was observed among included trials (*I^2 ^= *10.08%, *p*=0.339). Sensitivity analysis showed that heterogeneity may be derived from Takahashi’s data, so we consider the removal of the study by Takahashi et al. (25) (**Supplemental 1**). Subgroup analysis indicated that PCI significantly improved PFS in studies conducted in Western countries or in those with the proportion of males over 70.0% (**Supplemental 2**).

### Incidence of brain metastases

**Figure 4:**
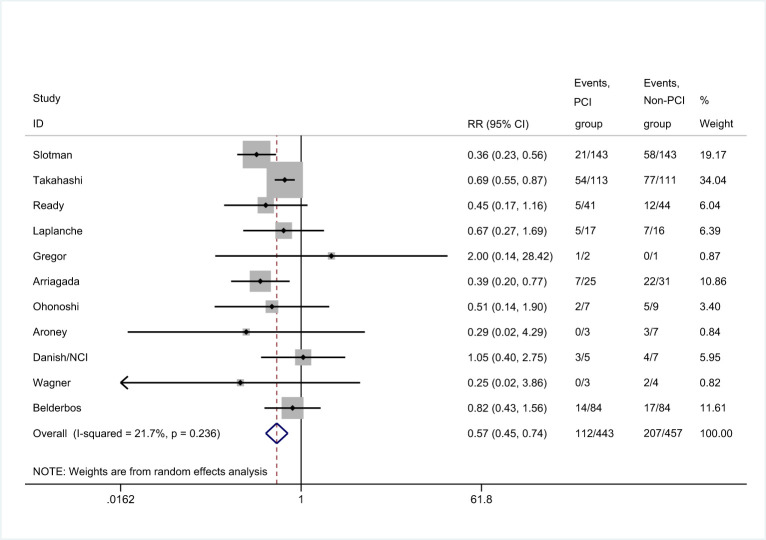
Effect of PCI on incidence of brain metastases in patients with extensive SCLC.

There were 11 trials that reported the incidence of brain metastases (8, 25, 26, 29–36). Meta-analysis showed that patients in PCI group had lower incidence of brain metastases than those in non-PCI group [RR=0.57, 95%CI (0.45, 0.74), *p*<0.001] (**Figure 4**). There was no significant heterogeneity considered among the studies. Sensitivity analysis showed that the conclusion was not affected by the exclusion of any of the study (**Supplemental 1**). Subgroup analysis found no significant difference in the incidence of brain metastases between PCI and non-PCI, when the mean age of included patients was less than 60.0 years-old (**Supplemental 2**).

### Survival rate

**Figure 5:**
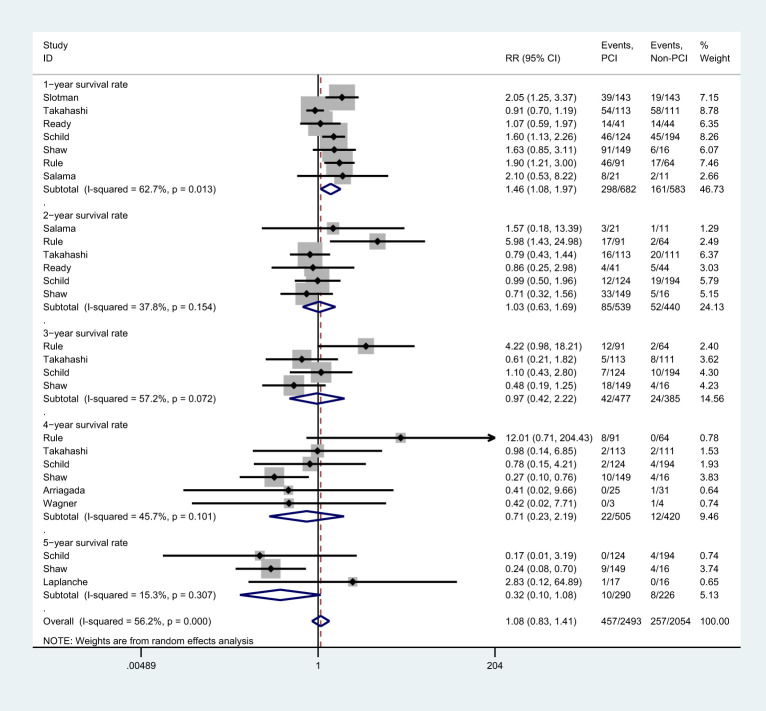
Effect of PCI on survival rates in different follow-up periods for patients with extensive SCLC.

There were 7 studies (8, 25–28, 37), 6 studies (25–28, 37), 4 studies (25, 27, 28,),6 studies (8, 27, 28, 31, 35,), and 3 studies (27–29) that reported 1-, 2-, 3-, 4-, and 5-year survival rate, respectively (**Figure 5**). Meta-analysis showed that PCI intervention did not improve the 2-year [RR=1.03, 95%CI (0.63, 1.69), *p*=0.154), 3-year [RR=0.97, 95%CI (0.42, 2.22), *p*=0.072], 4-year [RR=0.71, 95%CI (0.23, 2.19), *p*=0.101], and 5-year survival rate [RR=0.32, 95%CI (0.10, 1.08), *p*=0.307]. However, patients in PCI group had higher 1-year survival rate than those in non-PCI group [RR=1.46, 95%CI (1.08, 1.97), *p*=0.013]. The findings of the sensitivity analysis varied in 1-, 4-, and 5-year survival rates after excluding individual trials (**Supplemental 1**). Subgroup analysis showed that studies conducted in Western countries, studies with the proportion of male participants over 70.0%, and those with low-quality reported more increased 1-year survival rate. It also indicated no significant difference in 2- and 3-year survival rates between PCI and non-PCI group. Studies published before 2000, those conducted in Western countries, and those with lower JADAD scores reported lower 4-year survival rate. Finally, studies with more than 100 patients included or those with lower JADAD scores reported lower 5-year survival rate (**Supplemental 2**).

### Adverse events

All reported adverse events are summarized in **Table 2**. Patients in PCI group had higher risk of fatigue [RR=1.84, 95%CI (1.23, 2.73) *p*=0.003], dermatitis [RR=7.68, 95%CI (2.37, 24.90), *p*=0.001], anorexia [RR=2.22, 95%CI (1.44, 3.43), *p*<0.001], nausea [RR=3.84, 95%CI (1.93, 7.63), *p*<0.001], vomiting [RR=8.38, 95%CI (1.07, 65.84), *p*=0.043], malaise [RR=1.59, 95%CI (1.04, 2.44), *p*=0.034], and cognitive impairment [RR=2.24, 95%CI (1.09,4.62), *p*=0.028], compared with those in non-PCI group. There was no significant difference in the risk of alopecia, headache, dizziness, lethargy, muscle weakness, impaired role functioning, and impaired emotional functioning between the two groups.

**Table 2:** The summary results for adverse events.

Outcomes	Reference	RR and 95% CI	P value
Alopecia	8, 26	1.34 (0.90-2.00)	0.153
Fatigue	8	1.84 (1.23-2.73)	0.003
Dermatitis	26	7.68 (2.37-24.90)	0.001
Headache	26	2.44 (0.65-9.20)	0.187
Anorexia	26	2.22 (1.44-3.43)	<0.001
Nausea	26	3.84 (1.93-7.63)	<0.001
Vomiting	26	8.38 (1.07-65.84)	0.043
Dizziness	26	2.79 (0.76-10.25)	0.122
Malaise	26	1.59 (1.04-2.44)	0.034
Lethargy	26	2.79 (0.76-10.25)	0.122
Muscle weakness	26	1.05 (0.38-2.88)	0.929
Role functioning impairment	8	1.46 (0.93-2.29)	0.099
Cognitive impairment	8	2.24 (1.09-4.62)	0.028
Emotional functioning impairment	8	1.75 (0.90-3.43)	0.101

### Publication bias

Egger`s test and Begg`s test showed no significant publication bias in OS, PFS, incidence of brain metastases, and 1-, 2-, 3-, 4-, and 5-year survival rates (**Table 3**). The results did not reverse after adjustment for publication bias using the trim-and-fill method (38).

**Table 3:** Publication bias for all investigated outcomes.

Outcomes	P value for Egger test	P value for Begg test
OS	0.492	0.462
PFS	0.764	1.000
Brain metastases	0.550	1.000
1-year survival rate	0.317	0.806
2-year survival rate	0.999	1.000
3-year survival rate	0.800	1.000
4-year survival rate	0.373	1.000
5-year survival rate	0.607	0.296

## Discussion

In this study, 15 RCTs that evaluated the efficacy and safety of PCI in the treatment for patients with extensive stage SCLC were included, with a total of 1623 patients, and the results showed that PCI did not contribute to longer OS or PFS in the patients, compared with non-PCI. However, PCI significantly reduced the incidence of brain metastases. PCI did not affect the 2-, 3-, 4- and 5-year survival rates, but reduced the 1-year survival rate. PCI also showed to be associated with higher risk of adverse events including fatigue, dermatitis, anorexia, nausea, vomiting, malaise, and cognitive impairment in patients with extensive SCLC. The therapeutic effect of PCI might be subjected to different countries, sample size, age, gender, and study quality.

A previous study suggested that PCI significantly improved the survival rate, disease-free survival, and incidence of brain metastases in SCLC patients with complete remission (13). Another important meta-analysis indicated that PCI could improve the survival and decrease the incidence of brain metastases in SCLC patients, while it only consisted of 5 RCTs involving both limited and extensive stage SCLC patients (14), and the therapeutic effects on extensive stage SCLC patients with specific characteristics were not analyzed (13, 14). Schild et al. conducted a qualitative review examining the evidence and provided recommendations for the role of PCI in extensive stage SCLS patients, but did not perform any quantitative analyses (39). Therefore, this meta-analysis was exclusively focused on extensive stage SCLC to evaluate the potential efficacy and safety of PCI.

There were no significant differences in the improvements of OS and PFS between PCI group and non-PCI group. However, the results were inconclusive and needed to be further validated by large-scale trials. Furthermore, several studies included in our meta-analysis reported inconsistent results. Slotman et al. indicated that PCI could reduce the risk of symptomatic brain metastases and improve OS and PFS. They illustrated a greater impact of PCI for patients with extensive stage SCLC than for those with limited stage SCLC (8). A study conducted by Schild et al. indicated that PCI could prolong the survival in patients with both limited and extensive stage SCLC. They also found that PCI resulted in higher clinical response of chemotherapy and thoracic radiation therapy (9). The inconsistent findings of this study with previous studies could be related to the differences in the proportion of patients receiving subsequent treatment strategies.

We found that PCI could reduce the risk of brain metastases, which was consistent with previous studies (13, 14). A Cochrane review indicated that the incidence of brain metastases was reduced by 77% in patients receiving PCI. However, it was not assessed whether this effect would be different among specific subpopulations (13). On the other hand, the study conducted by Zhang et al. only included two studies to assess the incidence of brain metastases so that the results might be varied (14). Several included studies also reported inconsistent results (26, 29, 30, 32–35). A possible reason could be that the sample size was too small to reveal the clinical benefit, especially if the event rates were lower than expected. This could lead to broad confidence intervals resulting in no significantly statistical differences.

The results showed that PCI improved 1-year survival, although it had no significant effect on 2-, 3-,4 or 5-year survival. This is consistent with other studies that 1-year survival was significantly higher in the PCI group than in the control group (37.1% vs. 27.1%; Human Resources:0.87; 95% confidence interval:0.80 0.95; P = 0.002) (12). It might be that the median survival time of most patients with extensive stage SCLC is less than 1 year (5), resulting in more 1-year survival events than in the other follow-up periods, and a significant difference that was easier to detect due to the higher statistical power. Additionally, more than half of the included participants died within 1 year after recruited in the study, which might result in no significant differences in the 2-, 3-,4 or 5-year survival rates between the two groups. Subgroup analysis showed that region, sample size, mean age, proportion of included male patients, and study quality could affect the treatment effect of PCI in extensive stage SCLC patients. However, these significant differences between PCI and non-PCI groups were not observed in investigated outcomes (except for the incidence of brain metastases) in Eastern countries (25), and the conclusion might vary since smaller cohorts were included in such subsets. Moreover, ongoing treatment strategies, old age, and mutated genes might affect the therapeutic effect of PCI in extensive stage SCLC patients (40).

As expected, higher risk of adverse events was observed in patients receiving PCI, which would counteract the clinical benefits of PCI. Increased adverse events could compromise the quality of life in those patients, while the data on quality of life were rarely available in these studies. However, one study indicated that PCI had a negative impact on selected health-related quality of life scales and recommend that PCI should be offered to all responding extensive stage SCLC patients (41). The significantly increased risk of fatigue, dermatitis, anorexia, nausea, vomiting, malaise, and cognitive impairment in patients receiving PCI might also lead to poor quality of life. Alopecia and lethargy are the common toxic effects of PCI treatment, while our meta-analysis found no significant difference in the incidence of alopecia and lethargy between PCI and non-PCI groups, which might be related to the chemotherapies these patients were receiving, and only smaller trials reporting on these outcomes (2 for alopecia (8, 25) and 1 for lethargy (25)). Lastly, given the current disagreement among the involved clinical practitioners (42), ongoing randomized controlled trials also recruiting patients with ES-SCLC could help clarify which is the best therapeutic approach between whole brain radiotherapy and stereotactic radiosurgery for the management of overt brain metastases in cases without prior PCI (43).

This meta-analysis had several limitations. Firstly, subsequent chemotherapeutic regimens were different between PCI and non-PCI groups, and these data were not available in most included trials. Secondly, subgroup analysis based on dose/fractionation was not conducted since the mean dose and fractionation varied and lacked a standard cutoff value. Thirdly, publication bias was inevitable due to unpublished literatures were not searched so that the negative results could not be included. Therefore, the therapeutic effect of PCI for extensive stage SCLC might be overestimated in this study. Lastly, the analysis used pooled data (individual data were not available), which restricted us from performing a more detailed analysis and obtaining more comprehensive results.

In conclusion, PCI can reduce the incidence of brain metastases in extensive stage SCLC, and although it has no significant effect on overall survival, it improves 1-year survival in patients with extensive stage SCLC. Further, higher risk of adverse events was observed in patients receiving PCI. Based on these findings, we may make corresponding adjustments to the clinical management of patients with ES-SCLC. Before applying PCI, we should re-evaluate the patients according to their actual conditions, such as physical conditions and economic conditions, rather than blindly applying it. We will pay more attention to the 1-year survival advantages brought by PCI as well as the practical benefits of reducing the economic and psychological burdens of some patients. At present, the follow-up chemotherapy strategies for ES-SCLC patients after PCI are still controversial. Future studies require more clinical trials to help us re-evaluate the combination strategy of PCI and subsequent chemotherapy regimen for patients with extensive stage SCLC.

## Data availability statement

The original contributions presented in the study are included in the article/**Supplementary Material**. Further inquiries can be directed to the corresponding authors.

## Author contributions

ZW: Conceptualization and Writing of the first draft. LS, FC, LC, QY, XH, QF, WC: Review and Editing. PL and WL: Conceptualization and Review. All authors contributed to the article and approved the submitted version.
