# Supplementary material for: Prophylactic cranial irradiation for extensive stage small cell lung cancer: a meta-analysis of randomized controlled trials
Source: Front Oncol. 2023 May 17;13:1086290. doi: 10.3389/fonc.2023.1086290 (PMC10229841; doi:10.3389/fonc.2023.1086290)
Supplement: Supplementary file 1 [file Table_1.doc]

Table S1. sensitivity analysis for OS

| Excluding studies | HR and 95% CI | P value | I-square | P value for heterogeneity |
| --- | --- | --- | --- | --- |
| Slotman | 0.93(0.74-1.16) | 0.505 | 60.2 | 0.056 |
| Takahashi | 0.79(0.67-0.94) | 0.007 | 16.2 | 0.310 |
| Ready | 0.88(0.67-1.16) | 0.368 | 74 | 0.009 |
| Schild | 0.95(0.67-1.31) | 0.77 | 71.6 | 0.014 |
| Belderbos | 0.90(0.67-1.21) | 0.498 | 75.4 | 0.007 |
| Rule | 0.87(0.69-1.10) | 0.234 | 64.5 | 0.015 |
| Salama | 0.90(0.73-1.12) | 0.358 | 59.4 | 0.031 |

Table S2. sensitivity analysis for PFS

| Excluding studies | HR and 95% CI | P value | I-square | P value for heterogeneity |
| --- | --- | --- | --- | --- |
| Slotman | 0.86 (0.61-1.20) | 0.368 | 44.8 | 0.178 |
| Takahashi | 0.74 (0.60-0.92) | 0.006 | 0.0 | 0.703 |
| Ready | 0.86 (0.67-1.10) | 0.223 | 46.5 | 0.171 |
| Salama | 0.82(0.68-1.00) | 0.052 | 24.2 | 0.267 |

Table S3. sensitivity analysis for brain metastases

| Excluding studies | RR and 95% CI | P value | I-square | P value for heterogeneity |
| --- | --- | --- | --- | --- |
| Slotman | 0.66(0.55-0.80) | <0.001 | 0.0 | 0.721 |
| Takahashi | 0.51(0.39-0.67) | <0.001 | 1.8 | 0.422 |
| Ready | 0.58(0.44- 0.77) | <0.001 | 27 | 0.195 |
| Laplanche | 0.57(0.43-0.75) | <0.001 | 29.5 | 0.174 |
| Gregor | 0.57(0.44-0.73) | <0.001 | 25.2 | 0.211 |
| Arriagada | 0.60(0.47-0.78) | <0.001 | 18.6 | 0.271 |
| Ohonoshi | 0.57(0.440-0.76) | <0.001 | 29.3 | 0.175 |
| Aroney | 0.58(0.44-0.75) | <0.001 | 27.6 | 0.190 |
| Danish/NCI | 0.55(0.43-0.71) | <0.001 | 20.8 | 0.252 |
| Wagner | 0.58(0.44-0.75) | <0.001 | 27 | 0.195 |
| Belderbos | 0.54(0.41-0.72) | <0.001 | 25.6 | 0.208 |

Table S4. sensitivity analysis for 1-year survival rate

| Excluding studies | RR and 95% CI | P value | I-square | P value for heterogeneity |
| --- | --- | --- | --- | --- |
| Slotman | 1.23 (0.87-1.73) | 0.238 | 61.6 | 0.050 |
| Takahashi | 1.60 (1.26-2.03) | <0.001 | 0.0 | 0.448 |
| Ready | 1.43 (0.95-2.17) | 0.087 | 75.9 | 0.006 |
| Schild | 1.30 (0.85-2.01) | 0.230 | 69.5 | 0.020 |
| Shaw | 1.32 (0.89-1.97) | 0.169 | 74.7 | 0.008 |
| Rule | 1.39(1.00-1.93) | 0.050 | 62.1 | 0.021 |
| Salama | 1.44(1.05-1.97) | 0.023 | 68 | 0.008 |

Table S5. sensitivity analysis for 2-year survival rate

| Excluding studies | RR and 95% CI | P value | I-square | P value for heterogeneity |
| --- | --- | --- | --- | --- |
| Takahashi | 0.86 (0.53-1.38) | 0.524 | 0.0 | 0.819 |
| Ready | 0.83 (0.56-1.22) | 0.338 | 0.0 | 0.803 |
| Schild | 0.77 (0.49-1.20) | 0.249 | 0.0 | 0.962 |
| Shaw | 0.87 (0.53-1.33) | 0.511 | 0.0 | 0.886 |
| Rule | 0.84(.58-1.22) | 0.368 | 0 | 0.941 |
| Salama | 1.02（0.6-1.76） | 0.920 | 48.9 | 0.098 |

Table S6. sensitivity analysis for 3-year survival rate

| Excluding studies | RR and 95% CI | P value | I-square | P value for heterogeneity |
| --- | --- | --- | --- | --- |
| Takahashi | 0.73 (0.32-1.66) | 0.453 | 33.6 | 0.220 |
| Schild | 0.54 (0.26-1.10) | 0.088 | 0.0 | 0.738 |
| Shaw | 0.85 (0.42-1.74) | 0.666 | 0.0 | 0.429 |
| Rule | 0.70(0.39-1.23) | 0.215 | 0 | 0.462 |

Table S7. sensitivity analysis for 4-year survival rate

| Excluding studies | RR and 95% CI | P value | I-square | P value for heterogeneity |
| --- | --- | --- | --- | --- |
| Takahashi | 0.37 (0.16-0.83) | 0.016 | 0.0 | 0.743 |
| Schild | 0.37 (0.16-0.85) | 0.019 | 0.0 | 0.693 |
| Shaw | 0.71 (0.24-2.13) | 0.543 | 0.0 | 0.948 |
| Arriagada | 0.43 (0.20-0.93) | 0.032 | 0.0 | 0.542 |
| Wagner | 0.43 (0.20-0.93) | 0.032 | 0.0 | 0.543 |
| rule | 0.43(0.20-0.91) | 0.027 | 0 | 0.712 |

Table S8. sensitivity analysis for 5-year survival rate

| Excluding studies | RR and 95% CI | P value | I-square | P value for heterogeneity |
| --- | --- | --- | --- | --- |
| Schild | 0.55 (0.05-6.37) | 0.636 | 59.1 | 0.118 |
| Shaw | 0.66 (0.04-10.50) | 0.768 | 40.4 | 0.195 |
| Laplanche | 0.23 (0.09-0.63) | 0.004 | 0.0 | 0.811 |
