# Supplementary material for: Prophylactic cranial irradiation for extensive stage small cell lung cancer: a meta-analysis of randomized controlled trials
Source: Front Oncol. 2023 May 17;13:1086290. doi: 10.3389/fonc.2023.1086290 (PMC10229841; doi:10.3389/fonc.2023.1086290)
Supplement: Supplementary file 2 [file Table_2.docx]

Table S1. Subgroup analyses

| Outcomes | Group | HR or RR and 95%CI | P value | I^2^(%) and P value | P value between subgroups |
| --- | --- | --- | --- | --- | --- |
| OS | Publication year | | | | |
|  | 2000 or after | 0.87 (0.70-1.08) | 0.204 | 57.5/0.028 | - |
|  | Before 2000 | - | - | - |  |
|  | Country | | | | |
|  | Eastern | 1.27 (0.96-1.68) | 0.09 | - | 0.005 |
|  | Western | 0.78 (0.68-0.90) | 0 | 0/0.431 |  |
|  | Sample size | | | | |
|  | ≥ 100 | 0.88 (0.69-1.13) | 0.32 | 65.4/0.021 | 0.028 |
|  | < 100 | 0.80 (0.42-1.53) | 0.50 | 60.7/0.111 |  |
|  | Mean age (years) | | | | |
|  | ≥ 60.0 | 0.87 (0.70-1.08) | 0.204 | 57.5/0.028 | - |
|  | < 60.0 | - | - | - |  |
|  | Percentage male (%) | | | | |
|  | ≥ 70.0 | 1.27(0.96-1.68) | 0.09 | - | 0.204 |
|  | < 70.0 | 0.78 (0.68-0.90) | 0 | 0/0.431 |  |
|  | Study quality | | | | |
|  | 4-7 | 0.95 (0.72-1.24) | 0.694 | 62/0.032 | 0.204 |
|  | <4 | 0.74 (0.60-0.92) | 0 | 0/0.352 |  |
| PFS | Publication year | | | | |
|  | 2000 or after | 0.81(0.68-0.96) | 0.017 | 10.8/0.339 | - |
|  | Before 2000 | - | - | - |  |
|  | Country | | | | |
|  | Eastern | 0.98 (0.75-1.29) | 0.884 | - | 0.017 |
|  | Western | 0.73 (0.60-0.89) | 0.002 | 0.0/0.703 |  |
|  | Sample size | | | | |
|  | ≥ 100 | 0.86 (0.67-1.10) | 0.223 | 46.5/0.171 | 0.017 |
|  | < 100 | 0.67(0.47-0.95) | 0.027 | 0/0/785- |  |
|  | Mean age (years) | | | | |
|  | ≥ 60.0 | 0.81(0.68-0.96) | 0.017 | 10.8/0.339 | - |
|  | < 60.0 | - | - | - |  |
|  | Percentage male (%) | | | | |
|  | ≥ 70.0 | 0.98 (0.75-1.29) | 0.884 | - | 0.017 |
|  | < 70.0 | 0.73 (0.60-0.89) | 0.002 | 0.0/0.703 |  |
|  | Study quality | | | | |
|  | 4-7 | 0.82 (0.68-1.00) | 0.052 | 24.2/0.267 | 0.017 |
|  | <4 | 0.62(0.33-1.17) | 0.141 | - |  |
| Brain metastases | Publication year | | | | |
|  | 2000 or after | 0.50 (0.30-0.85) | 0.011 | 73.7/0.022 | 0.803 |
|  | Before 2000 | 0.56 (0.37-0.86) | 0.009 | 0.0/0.617 |  |
|  | Country | | | | |
|  | Eastern | 0.68 (0.55-0.86) | 0.001 | 0.0/0.665 | 0.011 |
|  | Western | 0.50 (0.37-0.68) | < 0.001 | 8/0.36 |  |
|  | Sample size | | | | |
|  | ≥ 100 | 0.58 (0.36-0.94) | 0.03 | 74.0/0.02 | 0.820 |
|  | < 100 | 0.54 (0.37-0.80) | 0.002 | 0.0/0.704 |  |
|  | Mean age (years) | | | | |
|  | ≥ 60.0 | 0.57 (0.41-0.79) | < 0.001 | 36.0/0.16 | 0.88 |
|  | < 60.0 | 0.60 (0.33-1.08) | 0.087 | 31.8/0.231 |  |
|  | Percentage male (%) | | | | |
|  | ≥ 70.0 | 0.65 (0.53-0.80) | < 0.001 | 0.0/0.457 | 0.049 |
|  | < 70.0 | 0.53 (0.31-0.91) | 0.02 | 45/0.14 |  |
|  | Study quality | | | | |
|  | 4-7 | 0.60 (0.44-0.80) | < 0.001 | 32.0/0.17 | 0.400 |
|  | <4 | 0.45 (0.25-0.80) | 0.007 | 0.0/0.495 |  |
| 1-year survival rate | Publication year | | | | |
|  | 2000 or after | 1.32 (0.89-1.97) | 0.169 | 74.7/0.008 | 0.387 |
|  | Before 2000 | 1.63 (0.85-3.11) | 0.139 | - |  |
|  | Country | | | | |
|  | Eastern | 0.91 (0.70-1.09) | 0.504 | - | 0.002 |
|  | Western | 1.60 (1.26-2.03) | < 0.001 | 0.0/0.448 |  |
|  | Sample size | | | | |
|  | ≥ 100 | 1.43 (0.95-2.17) | 0.087 | 75.9/0.006 | 0.671 |
|  | < 100 | 1.07 (0.59-1.97) | 0.819 | - |  |
|  | Mean age (years) | | | | |
|  | ≥ 60.0 | 1.32 (0.89-1.97) | 0.169 | 74.7/0.008 | - |
|  | < 60.0 | - | - | - |  |
|  | Percentage male (%) | | | | |
|  | ≥ 70.0 | 0.91 (0.70-1.19) | 0.504 | - | 0.007 |
|  | < 70.0 | 1.58 (1.16-2.15) | 0.003 | 24.5/0.266 |  |
|  | Study quality | | | | |
|  | 4-7 | 1.24 (0.73-2.10) | 0.428 | 76.3/0.015 | 0.041 |
|  | <4 | 1.61 (1.19-2.17) | 0.002 | 0.0/0.961 |  |
| 2-year survival rate | Publication year | | | | |
|  | 2000 or after | 0.87 (0.57-1.33) | 0.511 | 0.0/0.886 | 0.654 |
|  | Before 2000 | 0.71 (0.32-1.56) | 0.391 | - |  |
|  | Country | | | | |
|  | Eastern | 0.79 (0.43-1.44) | 0.433 | - | 0.837 |
|  | Western | 0.86 (0.53-1.38) | 0.524 | 0.0/0.819 |  |
|  | Sample size | | | | |
|  | ≥ 100 | 0.83 (0.56-1.22) | 0.338 | 0.0/0.803 | 0.955 |
|  | < 100 | 0.86 (0.25-2.98) | 0.810 | - |  |
|  | Mean age (years) | | | | |
|  | ≥ 60.0 | 0.87 (0.57-1.33) | 0.511 | 0.0/0.886 | - |
|  | < 60.0 | - | - | - |  |
|  | Percentage male (%) | | | | |
|  | ≥ 70.0 | 0.79 (0.43-1.44) | 0.433 | - | 0.817 |
|  | < 70.0 | 0.96 (0.52-1.74) | 0.884 | 0.0/0.846 |  |
|  | Study quality | | | | |
|  | 4-7 | 0.80 (0.46-1.37) | 0.418 | 0.0/0.900 | 0.876 |
|  | <4 | 0.86 (0.51-1.44) | 0.555 | 0.0/0.526 |  |
| 3-year survival rate | Publication year | | | | |
|  | 2000 or after | 0.85 (0.42-1.74) | 0.666 | 0.0/0.429 | 0.338 |
|  | Before 2000 | 0.48 (0.19-1.25) | 0.135 | - |  |
|  | Country | | | | |
|  | Eastern | 0.61 (0.21-1.82) | 0.379 | - | 0.848 |
|  | Western | 0.73 (0.32-1.66) | 0.453 | 33.6/0.220 |  |
|  | Sample size | | | | |
|  | ≥ 100 | 0.70 (0.39-1.23) | 0.215 | 0.0/0.462 | - |
|  | < 100 | - | - | - |  |
|  | Mean age (years) | | | | |
|  | ≥ 60.0 | 0.85 (0.42-1.74) | 0.666 | 0.0/0.429 | - |
|  | < 60.0 | - | - | - |  |
|  | Percentage male (%) | | | | |
|  | ≥ 70.0 | 0.61 (0.21-1.82) | 0.379 | - | 0.462 |
|  | < 70.0 | 1.10 (0.43-2.80) | 0.850 | - |  |
|  | Study quality | | | | |
|  | 4-7 | 0.61 (0.21-1.82) | 0.379 | - | 0.848 |
|  | <4 | 0.73 (0.32-1.66) | 0.453 | 33.6/0.220 |  |
| 4-year survival rate | Publication year | | | | |
|  | 2000 or after | 0.86 (0.24-3.08) | 0.820 | 0.0/0.862 | 0.162 |
|  | Before 2000 | 0.29 (0.11-0.74) | 0.010 | 0.0/0.933 |  |
|  | Country | | | | |
|  | Eastern | 0.98 (0.14-6.85) | 0.986 | - | 0.347 |
|  | Western | 0.37 (0.16-0.83) | 0.016 | 0.0/0.743 |  |
|  | Sample size | | | | |
|  | ≥ 100 | 0.44 (0.19-1.04) | 0.061 | 7.9/0.338 | 1.000 |
|  | < 100 | 0.41 (0.05-3.53) | 0.420 | 0.0/0.994 |  |
|  | Mean age (years) | | | | |
|  | ≥ 60.0 | 0.86 (0.24-3.08) | 0.820 | 0.0/0.862 | 0.365 |
|  | < 60.0 | 0.41 (0.02-9.66) | 0.580 | - |  |
|  | Percentage male (%) | | | | |
|  | ≥ 70.0 | 0.77 (0.15-4.04) | 0.760 | 0.0/0.644 | 0.400 |
|  | < 70.0 | 0.78 (0.15-4.21) | 0.775 | - |  |
|  | Study quality | | | | |
|  | 4-7 | 0.75 (0.15-3.80) | 0.733 | 0.0/0.632 | 0.419 |
|  | <4 | 0.36 (0.16-0.85) | 0.020 | 0.0/0.536 |  |
| 5-year survival rate | Publication year | | | | |
|  | 2000 or after | 0.17 (0.01-3.19) | 0.238 | - | 1.000 |
|  | Before 2000 | 0.55 (0.05-6.37) | 0.636 | 59.1/0.118 |  |
|  | Country | | | | |
|  | Eastern | - | - | - | - |
|  | Western | 0.32 (0.10-1.08) | 0.067 | 15.3/0.307 |  |
|  | Sample size | | | | |
|  | ≥ 100 | 0.23 (0.09-0.63) | 0.004 | 0.0/0.811 | 0.129 |
|  | < 100 | 2.83 (0.12-64.89) | 0.514 | - |  |
|  | Mean age (years) | | | | |
|  | ≥ 60.0 | 0.17 (0.01-3.19) | 0.238 | - | 0.307 |
|  | < 60.0 | 2.83 (0.12-64.89) | 0.514 | - |  |
|  | Percentage male (%) | | | | |
|  | ≥ 70.0 | 2.83 (0.12-64.89) | 0.514 | - | 0.307 |
|  | < 70.0 | 0.17 (0.01-3.19) | 0.238 | - |  |
|  | Study quality | | | | |
|  | 4-7 | 2.83 (0.12-64.89) | 0.514 | - | 0.129 |
|  | <4 | 0.23 (0.09-0.63) | 0.004 | 0.0/0.811 |  |
